# The Use of Life’s Essential 8 as a Prediction Model and Beyond∗

**DOI:** 10.1016/j.jacadv.2024.100944

**Published:** 2024-05-01

**Authors:** Frederick K. Ho, Fanny Petermann-Rocha

**Affiliations:** a School of Health and Wellbeing, University of Glasgow, Glasgow, United Kingdom; b Centro de Investigación Biomédica, Facultad de Medicina, Universidad Diego Portales, Santiago, Chile; c School of Cardiovascular and Metabolic Health, University of Glasgow, Glasgow, United Kingdom

**Keywords:** cardiovascular disease, Life Essential 8, Life Simple 7, mortality, risk prediction

**Figure:**
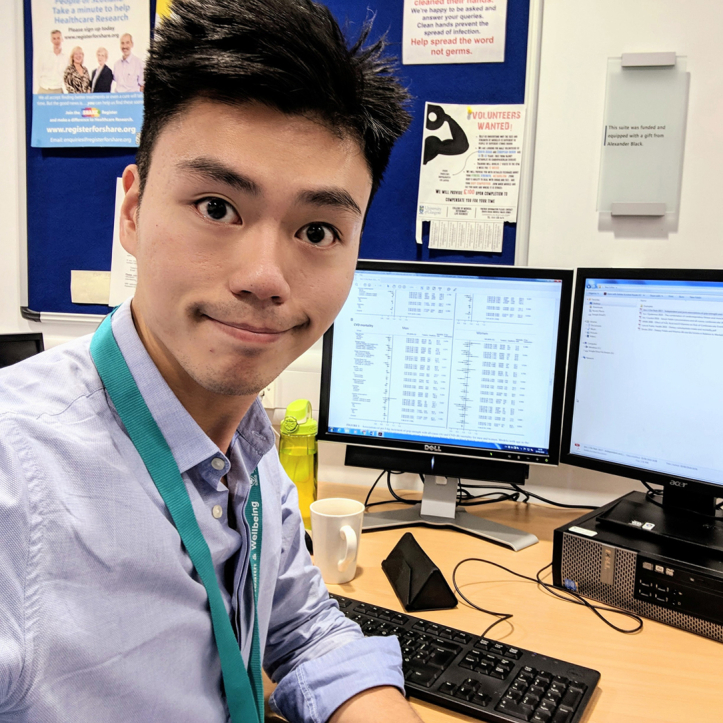

In 2010, the American Heart Association developed the Life’s Simple 7 (LS7) to promote positive cardiovascular health through seven predictors of heart health: not smoking, healthy weight, eating healthy, being physically active as well as promoting better blood pressure, cholesterol, and blood sugar levels. The LS7 marked an important paradigm shift from the conventional disease prevention model.^1^ Prospective studies investigating its role over adverse health outcomes highlighted that higher score levels (with a maximum of 14 points) were associated with a lower risk of these conditions.^2^, ^3^, ^4^ In 2022, the score was updated to become the Life’s Essential 8 (LE8) with the addition of sleep as a component and a refinement of the original seven domains as well as a maximum score of 100.^5^ Since its update, the novel construct attracted vast interest from the scientific community. One well-studied area is on whether the LS7 and LE8 were actually associated with prospective cardiovascular^6^, ^7^, ^8^ and noncardiovascular outcomes, such as dementia,^9^ chronic kidney disease,^10^ and cancer.^11^ Since many of these health outcomes have shared lifestyle (eg physical activity) and physical (eg body mass index) correlates, it is not surprising that most studies found that a higher score in LS7 or LE8 is associated with lower health risk.

Another research area around LS7 and LE8 is on their use in prediction. For instance, there have been studies that explored the LS7 and LE8’s prediction utility on subclinical coronary atherosclerosis,^12^ primary^13^ and secondary cardiovascular outcomes,^14^ and various other outcomes. However, one important question around LS7 and LE8’s use in prediction is how it is compared to the existing prediction models, namely the American Heart Association pooled cohort question (PCE).

To answer that question in this issue of *JACC: Advances*, Shetty et al^15^ pooled the National Health and Nutrition Examination Survey carried out between 2007 and 2018 and examined the mortality risk discrimination of LS7 and LE8 in 21,721 participants aged above 18 years. They found strong C statistics in predicting both all-cause mortality (LS7: 0.819; LE8: 0.823) and cardiovascular mortality (LS7: 0.883; LE8: 0.887) in the overall adult population, even though the differences between the two versions were marginal, consistent with previous studies that examined incident outcomes.^9^^,^^13^ They also compared the LS7 and LE8 with PCE among participants aged 40 to 79 years, for whom the PCE was designed. The C statistics of LS7 (0.674) and LE8 (0.681), however, were much weaker when applied to people aged 40 to 79 years. Importantly, these C statistics were also substantially worse than the PCE (0.756).

Even though PCE was primarily developed for incident cardiovascular disease, the findings of this study illustrated its superiority in predicting mortality compared to the LS7 and LE8. We would argue that this vast difference in C statistics should not be a surprise. The PCE was developed and designed for predicting atherosclerotic cardiovascular disease,^16^ a major contributor to all-cause and cardiovascular mortality. In contrast, the LS7 and LE8 were designed to measure a positive construct—cardiovascular health—for motivating lifestyle changes. The design of LS7 and LE8 selected factors that are easily measurable, actionable, and the target health outcome is cardiovascular disease-free survival and quality of life.^1^^,^^5^ Even though LS7 and LE8 were repeatedly found to be moderately predictive of cardiovascular and other health outcomes, prediction was not a primary concern in their design. This is also evident from the LS7 and LE8 scoring system. Instead of using raw, continuous values with model-derived coefficients (or weightings) for each component as in the PCE,^16^ both the LS7 and LE8 apply equal weighting of all its components.^1^^,^^5^ That is to assume all components would have equal importance on cardiovascular health. It also did not include other important, but nonmodifiable, factors such as age and sex. As such, Shetty et al^15^ pointed out an important conclusion: *“future research should redirect efforts away from assessing the risk prediction value of these tools and toward using these scores to characterize and track cardiovascular health as intended”*.^15^ Therefore, given the focus of LS7 and LE8, it is unlikely that they would have better predictions than purposely built tools.

It is, however, not to say that research on LS7 and LE8 is not worth pursuing. To the contrary, several important areas are underexplored. For example, being a positive construct to quantify cardiovascular health, studies could focus on the surveillance of overall cardiovascular health and its components, as in a recent meta-analysis,^17^ and whether a particular component and/or a population subgroup is of worse progress. These will be very useful in informing interventions. It might also be interesting to examine cardiovascular health using the adapted version of LE8 in childhood^18^ and track its trajectories over the life course to explore what affects cardiovascular health over different time points. Remarkably, as a simple tool, the use of LE8 in informing and as a part of intervention should be explored.^19^ Because people with lower LE8 scores were at exponentially higher risk of cardiovascular outcomes, it was estimated that a targeted intervention focusing on people with the lowest score would be more effective than a blanket population intervention.^6^ However, further economic analysis should be conducted to examine what interventions could bring the most benefits. Last but not least, because of LE8’s association with both cardiovascular and noncardiovascular outcomes, it would be interesting to explore whether LE8, or a modified version of it, could be regarded as a proxy for general health and well-being during the whole life cycle.

## Funding support and author disclosures

The authors have reported that they have no relationships relevant to the contents of this paper to disclose.

## References

[1] Lloyd-Jones D.M., Hong Y., Labarthe D. (2010). Defining and setting national goals for cardiovascular health promotion and disease reduction: the American Heart Association’s strategic Impact Goal through 2020 and beyond. Circulation.

[2] Isiozor N.M., Kunutsor S.K., Voutilainen A., Kauhanen J., Laukkanen J.A. (2021). Life's Simple 7 and the risk of stroke in Finnish men: a prospective cohort study. Prev Med.

[3] Uijl A., Koudstaal S., Vaartjes I. (2019). Risk for heart failure: the opportunity for prevention with the American Heart Association’s Life’s Simple 7. J Am Coll Cardiol HF.

[4] Ahmed A., Pinto Pereira S.M., Lennon L., Papacosta O., Whincup P., Wannamethee G. (2020). Cardiovascular health and stroke in older british men: prospective findings from the British Regional Heart Study. Stroke.

[5] Lloyd-Jones D.M., Allen N.B., Anderson C.A. (2022). Life’s essential 8: updating and enhancing the American Heart Association’s construct of cardiovascular health: a presidential advisory from the American Heart Association. Circulation.

[6] Petermann-Rocha F., Deo S., Celis-Morales C. (2023). An opportunity for prevention: associations between the Life's Essential 8 score and cardiovascular incidence using prospective data from UK Biobank. Curr Probl Cardiol.

[7] Wu S., Wu Z., Yu D. (2023). Life’s Essential 8 and risk of stroke: a prospective community-based study. Stroke.

[8] Sun J., Li Y., Zhao M. (2023). Association of the American Heart Association’s new “Life’s Essential 8” with all-cause and cardiovascular disease-specific mortality: prospective cohort study. BMC Med.

[9] Petermann-Rocha F., Deo S., Lyall D. (2023). Association between the AHA Life's Essential 8 score and incident all-cause dementia: a prospective cohort study from UK Biobank. Curr Probl Cardiol.

[10] Ruan Y.-X., Wu M.-X., Gao J.-W. (2023). AHA Life’s Essential 8 and new-onset CKD: a prospective cohort study from the UK Biobank. Clin Exp Nephrol.

[11] Makarem N., Dinh V., Hosalli R., Aggarwal B.A., German C.A., Mullachery P. (2023). Life’s essential 8 and mortality risk: associations of an Enhanced cardiovascular health construct with all-cause, cardiovascular, and cancer mortality in US adults from the 2011-2018 national health and Nutrition Examination Survey. Circulation.

[12] Herraiz-Adillo Á., Higueras-Fresnillo S., Ahlqvist V.H. (2024). Life’s essential 8 and life’s simple 7 in relation to coronary Atherosclerosis: results from the population-based SCAPIS project. Mayo Clin Proc.

[13] Howard G., Cushman M., Blair J. (2023). Comparative discrimination of Life’s simple 7 and Life’s essential 8 to Stratify cardiovascular risk: is the added Complexity worth it?. Circulation.

[14] Gao X., Ma X., Lin P. (2023). Predictive value of cardiovascular health score for health outcomes in Patients with PCI: Comparison between Life’s simple 7 and Life’s essential 8. Int J Environ Res Publ Health.

[15] Shetty N., Gaonkar M., Patel N., Li P., Arora G., Arora P. (2024). Association of Life’s essential 8 and simple 7 scores with mortality: comparison with pooled cohort Equation. JACC: Adv.

[16] Goff D.C., Lloyd-Jones D.M., Bennett G. (2014). 2013 ACC/AHA guideline on the assessment of cardiovascular risk: a report of the American College of Cardiology/American heart association Task Force on Practice Guidelines. J Am Coll Cardiol.

[17] López-Bueno R., Núñez-Cortés R., Calatayud J. (2023). Global prevalence of cardiovascular risk factors based on the Life's Essential 8 score: an overview of systematic reviews and meta-analysis. Cardiovasc Res.

[18] Perng W., Aris I.M., Slopen N. (2023). Application of Life’s Essential 8 to assess cardiovascular health during early childhood. Ann Epidemiol.

[19] Thomas V.E., Metlock F.E., Hines A.L., Commodore-Mensah Y., Brewer L.C. (2023). Community-based interventions to address disparities in cardiometabolic diseases among minoritized racial and ethnic groups. Curr Atherosclerosis Rep.

